# Spatial accessibility to basic public health services in South Sudan

**DOI:** 10.4081/gh.2017.510

**Published:** 2017-05-11

**Authors:** Peter M. Macharia, Paul O. Ouma, Ezekiel G. Gogo, Robert W. Snow, Abdisalan M. Noor

**Affiliations:** 1 Kenya Medical Research Institute/Wellcome Trust Research Programme, Nairobi, Kenya; 2 Centre for Tropical Medicine and Global Health, Nuffield Department of Clinical Medicine, University of Oxford, Oxford, UK

**Keywords:** South Sudan, Health facilities, Spatial accessibility

## Abstract

At independence in 2011, South Sudan’s health sector was almost non-existent. The first national health strategic plan aimed to achieve an integrated health facility network that would mean that 70% of the population were within 5 km of a health service provider. Publically available data on functioning and closed health facilities, population distribution, road networks, land use and elevation were used to compute the fraction of the population within 1 hour walking distance of the nearest public health facility offering curative services. This metric was summarised for each of the 78 counties in South Sudan and compared with simpler metrics of the proportion of the population within 5 km of a health facility. In 2016, it is estimated that there were 1747 public health facilities, out of which 294 were non-functional in part due to the on-going civil conflict. Access to a service provider was poor with only 25.7% of the population living within one-hour walking time to a facility and 28.6% of the population within 5 km. These metrics, when applied sub-nationally, identified the same high priority, most vulnerable counties. Simple metrics based upon population distribution and location of facilities might be as valuable as more complex models of health access, where attribute data on travel routes are imperfect or incomplete and sparse. Disparities exist in South Sudan among counties and those with the poorest health access should be targeted for priority expansion of clinical services.

## Introduction

Access to health services is important for a population’s health status and the measurement of its variability is critical to effective allocation of national health resources. Accessibility can be measured on the basis of financial costs to clients, availability of the required resources at health facilities, quality of health care, acceptability and geographic accessibility (Penchansky and Thomas, 1981; Guagliardo, 2004). The latter is the most commonly measured as it is seen as an easily quantifiable and interpretable measure for policy decisions at national and global scales. Spatial accessibility can be defined using indices such as the health facility-to-population ratio or the estimated proportion of population within a specified distance or travel time to health facilities. Clearly, any analysis of spatial accessibility depends critically upon the availability of spatially defined data on the location of health facilities and population (Guagliardo, 2004).

According to the World Health Organization (WHO), child and maternal mortality remain among the highest in the world in South Sudan (WHO, 2014; Valadez *et al*., 2015). Coincidentally, access to health care is considered one of the poorest in Africa (WHO and World Bank, 2015). The war for independence was one of the longest in the continent (Cometto *et al*., 2010). At independence in July 2011, public services, especially in the health sector, were virtually non-existent. In an attempt to rapidly improve the situation, the Health Sector Development Programme (HSDP) for 2012-2016 was launched with the ambitious target of ensuring 70% of the population to have access to health care by 2015, (Government of South Sudan Ministry of Health, 2012). Civil war re-erupted within South Sudan in December 2013.

In 2012, it was estimated that effective coverage of health services was poor. The World Bank, GSS-MoH and others have estimated that between 40-45% of the population are settled within 5 km of a health service provider (Government of South Sudan Ministry of Health, 2012; Downie, 2012; Shabalina, 2014; World Bank, 2014). Other reports suggest that between 25-33% of the population have access to basic and adequate health services (Vogt *et al*., 2011; WHO, 2009; Cometto *et al*., 2010). These aggregated national estimates are hard to compare as each has a slightly different meaning and few are accompanied with what data sources used to compute the metrics. Importantly none of these national summaries allow for a more effective understanding of sub-national health access, necessary for planning of effective, equitable resource alocation.

In this study, publically available data on health services, population and transport networks are used within a geographic information system (GIS) to develop spatial models that provide a variety of updated spatial health access metrics nationally and sub-nationally for South Sudan.

## Materials and Methods

### Country and health service context

**Figure 1:**
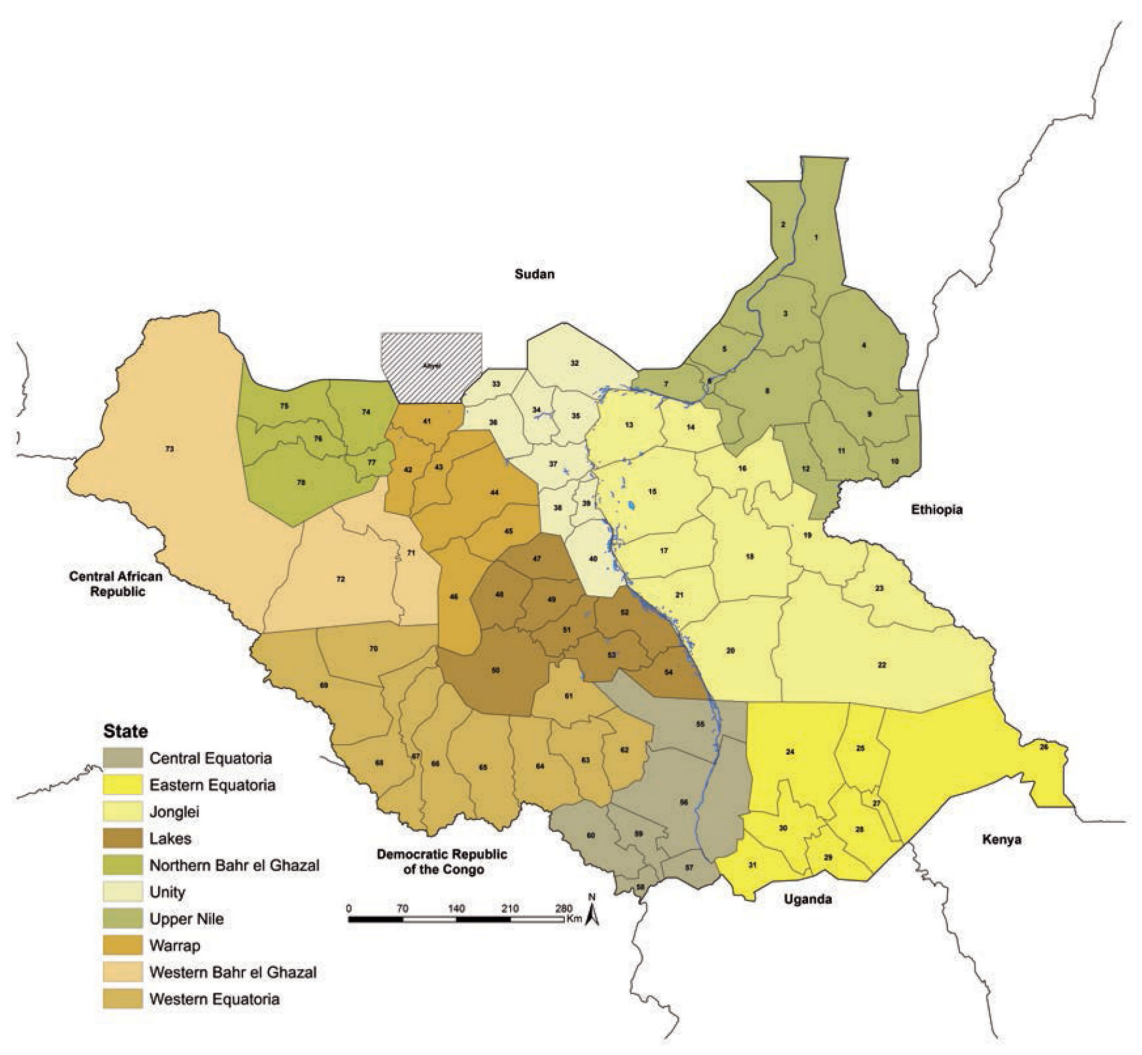
Map of South Sudan counties (n=78) excluding Abyei disputed area across 10 States. County codes are as follows: Upper Nile: Renk (1), Manyo (2), Melut (3), Maban (4), Fashoda (5), Malakal (6), Panyikang (7), Baliet (8), Longochuk (9), Maiwut (10), Luakpiny/Nasir (11), Ulang (12); Jonglei: Fangak (13), Canal/Pigi (14), Ayod (15), Nyirol (16), Duk (17), Uror (18), Akobo (19), Bor South (20), Twic East (21), Pibor (22), Pochalla (23); Eastern Equatoria: Lafon (24), Kapoeta North (25), Kapoeta East (26), Kapoeta South (27), Budi (28), Ikotos (29), Torit (30), Magwi (31); Unity: Pariang (32), Abiemnhom (33), Rubkona (34), Guit (35), Mayom (36), Koch (37), Mayendit (38), Leer (39), Panyijiar (40); Warrap: Twic (41), Gogrial West (42), Gogrial East (43), Tonj North (44), Tonj East (45), Tonj South (46); Lakes: Rumbek North (47), Cueibet (48), Rumbek Centre (49), Wulu (50), Rumbek East (51), Yirol East (52), Yirol West (53), Awerial (54); Central Equatoria: Terekeka (55), Juba (56), Kajo-keji (57), Morobo (58), Lainya (59), Yei (60); Western Equatoria: Mvolo (61), Mundri East (62), Mundri West (63), Maridi (64), Ibba (65), Yambio (66), Nzara (67), Ezo (68), Tambura (69), Nagero (70); Western Bahr el Ghazal: Jur River (71), Wau (72), Raga (73); and Northern Bahr el Ghazal: Aweil East (74), Aweil North (75), Aweil West (76), Aweil South (77) and Aweil Centre (78).

South Sudan has an approximate area of 640,000 km^2^ and is divided into 10 states and 79 counties (including the disputed territory of Abyei), and they represent the first and second levels of administration, respectively (Figure 1) (Government of South Sudan Ministry of Health, 2011, 2012) and covered an estimated population of 12 million people in 2015 according to Southern Sudan Centre for Census Statitistics and Evaluation (SSCCSE) (Southern Sudan Centre for Census Statitistics and Evaluation, 2010; Government of South Sudan Ministry of Health, 2012). Approximately 90% of the population live in rural areas (Southern Sudan Centre for Census Statitistics and Evaluation, 2010; Government of South Sudan Ministry of Health, 2012).

The formal health services in South Sudan are provided mainly by the Government supported by non-governmental organizations (NGOs) and faith based organizations (FBOs) (Government of South Sudan Ministry of Health, 2013). The public health sector is structured along four tiers: primary health care units (PHCUs), primary health care centres (PHCCs), county hospitals (CHs), state hospitals (SHs) and teaching hospitals (THs) (Government of South Sudan Ministry of Health, 2012). PHCUs are the first level of primary care and provide basic preventive, promotive and curative services and expected to serve a population of 15,000. PHCCs, aimed at serving a population of 50,000, are the immediate reference facilities for the PHCUs, providing all the services provided by a PHCU but in theory additional services covering diagnostic laboratory, maternity and inpatient care. The CHs are referral facilities for PHCCs and provide emergency services aimed at serving a maximum population of 300,000 people, while SHs are designed to serve a population of 500,000 people. CHs and SHs represent the secondary health care level while THs provide tertiary care. Although, according to the national policy health care is decentralised with governance at the national, state and county levels (Government of South Sudan Ministry of Health, 2011), the national Government still retains most regulatory, budgetary and commodity supply responsibilities. The private sector is in a nascent stage with a few private clinics, mostly in urban areas, but they are poorly equipped (Cornelis and Ron, 2006).

### Spatial databases

#### Health facilities

A health facility database was obtained from the Humanitarian Data Exchange Portal (HDX) of the United Nations Office for the Coordination of Humanitarian Affairs (UN-OCHA) (UN-OCHA, 2016a) developed originally from a mapping exercise undertaken by the MoH and the World Bank in 2009 and regularly updated by UN-OCHA. The original database had 1877 health facilities, with the majority of these facilities (78.5%) already mapped using global positioning system (GPS) receivers. For those that had not been spatially positioned, coordinates were derived through geocoding of place or village names, using a settlement dataset of South Sudan (UN OCHA, 2016b) and online sources including Google Earth (http://www.google.co.uk/intl/en_uk/earth/) and Geo-names (http://www.geonames.org/search.html?q=&country=SS=). Duplicate facilities were removed, those labelled non-operational due to conflict, destruction and lack of resources, private, or reserved for specialised care or security forces were also removed. The final database covered all NGO, FBO and Government services capable of offering the basic preventive, promoter and curative health services to the general public.

#### Population

**Figure 2:**
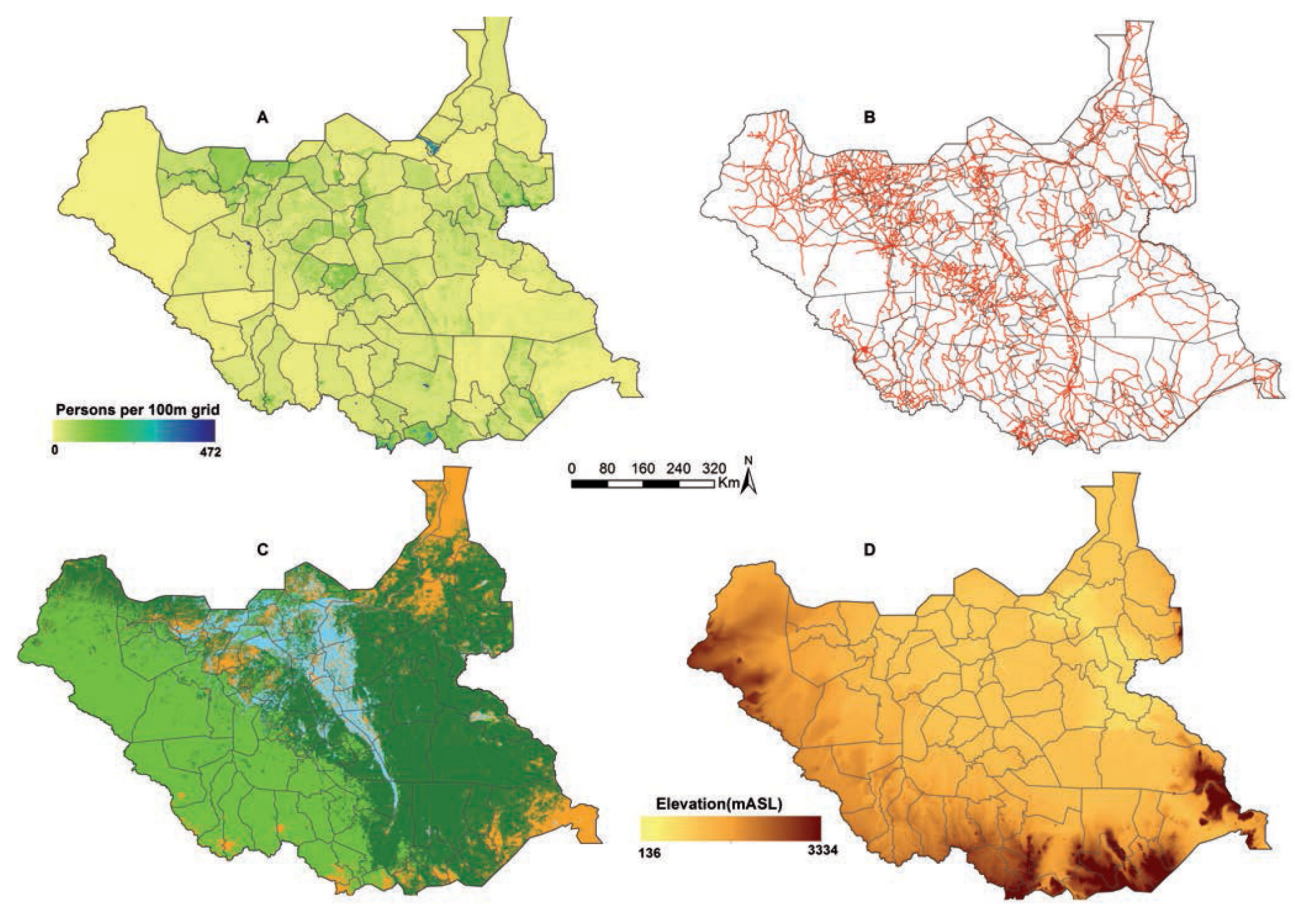
A) Modelled population distribution at 100×100 m spatial resolution; B) road network; C) major land cover classes: shrub land dark green (46.5%), tree cover (open deciduous broadleaved) green (35.1%), shrub/herbaceous cover flooded with fresh/saline/brackish water blue (7.5%), and others brown (10.9%); D) altitude measured by digital elevation model [metres above the mean sea level (m asl)] with an increase in elevation from light yellow (136 m asl) to dark brown (3334 m asl).

Critical to the modelling of spatial accessibility to services is a reliable understanding of the distribution and density of the populations they serve. The last census in South Sudan was undertaken in 2008 and data are not available below county levels. To overcome this low-resolution population data, WorldPop (http://www.worldpop.org.uk/) has developed spatial disaggregation techniques to redistribute population counts to finer spatial resolutions (Linard *et al*., 2012; Stevens *et al*., 2015). These models for South Sudan have reallocated populations within census units by disaggregating population count data from the 79 counties in 2008 to a 100 m spatial resolution grid using Land cover class population densities derived from 30m Landsat satellite imagery (http://landsat.visibleearth.nasa.gov/) as weights using dasymetric modelling techniques (Mennis, 2009; Linard *et al*., 2011). Each grid was then projected to match the UN national population estimates for the year 2015 (United Nations Population Division, 2015). Population distribution, according to the WorldPop predictions highlights the over-dispersion of settlements in South Sudan, with the highest concentration of people in southern most counties in Central Equatoria State, and in the states of Warrap and Northern Bahr el Ghazal (Figure 2A).

#### Road network

People mostly travel on road networks to reach services rather than along straight lines (Euclidean) from their homes to the point of service provision. Therefore, a road surface was developed as a composite of three online sources: Global Roads Open Access Data Set, assembled mostly from Vector Smart Map Level 0 (VMap0), Edition 5 for the period 1980 – 2010 (Center for International Earth Science Information Network, 2013); the HDX portal, which sourced data from the World Food Programme and last updated in December 2012 (UN OCHA, 2016c); and from Open Street Map which is mapped and maintained by volunteers all over the world (OpenStreetMap, 2015). The three road network data were merged and duplicates removed in ArcGIS, version 10.1 (ESRI Inc., Redlands, CA, USA). The resultant database of the road network was exported to Google Earth and all visually identifiable roads and footpaths digitised in areas where they had not been captured. The road network across the country is patchy, of 17,000 km, only 200 km is paved road (World Bank, 2016). During the dry season most of unpaved roads are only accessible by allterrain vehicles, while during the rainy season most roads are impassable (UN OCHA, 2014a) (Figure 2B).

#### Land cover and use

Satellite-derived information is available on land cover and land use, identifying properties of the geographical space people need to traverse. Data were obtained from the GlobCover (http://maps.elie.ucl.ac.be/CCI/viewer/) for the 2010 epoch (2008-2012) that used the processed 300-m and 1-km spatial resolution Medium Resolution Spectrometer (MERIS) (https://earth.esa.int/web/guest/missions/esa-operational-eo-missions/envisat/instruments/meris) and SPOT-VEGETATION (http://www.vgt.vito.be/) sensors respectively. The hierarchical classification used is based on United Nations Land Cover Classification System of the Food and Agricultural Organization (FAO) (FAO, 2000). The topography of the country is less varied with shrub land and broadleaved, deciduous, open tree cover dominant across most parts with a stretch of shrub/herbaceous cover, brackish flooded water along the edges of River Nile (Figure 2C).

#### Digital elevation model

One impedance to walking travel times is slope (Tobler, 1993). To assemble elevations above sea level the Advanced Space borne Thermal Emission and Reflection Radiometer (ASTER) Global Digital Elevation Model Version 2 (GDEM V2) (https://asterweb.jpl.nasa.gov/gdem.asp) was used at a spatial resolution of 30 m (Figure 2D).

### Analysis of spatial accessibility

Two measures of spatial accessibility were computed: the proportion of population by county within 5 km Euclidean distance to any public health facility and the proportion of population by county within one hour travel time to any public health facility. Sub-national descriptions of populations to facility ratios are less valuable as people seek treatment across county boundaries, so while useful as a measure of national health access, for policy targets and international comparisons, it is less valuable for sub-national mapping of vulnerability.

#### Euclidean distances

The location of each health facility was then used to develop a 100×100 m spatial resolution surface of Euclidean distance to the nearest health facility using the *Euclidean Distance Tool* in ArcGIS. The proportion of population within 5-km Euclidean distance was extracted from the gridded population surface using the *Zonal statistics Tool* in ArcGIS for each county.

#### Travel time

The topologically defined road network was converted into raster surface of roads matching that of the land cover surface. The *rasterised* road surface and the land cover classes were then assigned travel time equivalent to the number of hours taken to cross each cell based on the type. All the roads were assigned a walking speed of 5 km/h (Noor *et al*., 2006; Ray and Ebener, 2008), while different land cover features were assigned different recommended walking speeds ranging from 1 km/h across tree cover and flooded areas to 5 km/h across shrub land (Alegana *et al*., 2012). Slope derived from digital elevation model (DEM) was used to calculate the actual surface distance covered between contiguous cells and to adjust the walking speed by decreasing the up-slope and down-slope speed with increase in slope, while slightly increasing the speed for a slightly negative slope when walking down-slope using the *Path Distance Tool* of ArcGIS. For travel time analysis, only walking distances to the nearest health facilities were computed, based upon the fact that 90% of the population are rural and have been reported to have very low access to vehicular transport because of the very poor road infrastructure (Downie, 2012; GSS-MOH, 2012; UN OCHA, 2012). The walking time to the health facilities was estimated from each population grid by identifying the shortest distance to the nearest health facility. The total walking time was then computed by adding up the time needed to cross-adjoining cells to the nearest health facility using the Path *Distance Tool* of ArcGIS. The proportion of population within one hour was extracted using the *Zonal Statistic Tool* of ArcGIS at the county level.

## Results

### Health service distribution

**Figure 3:**
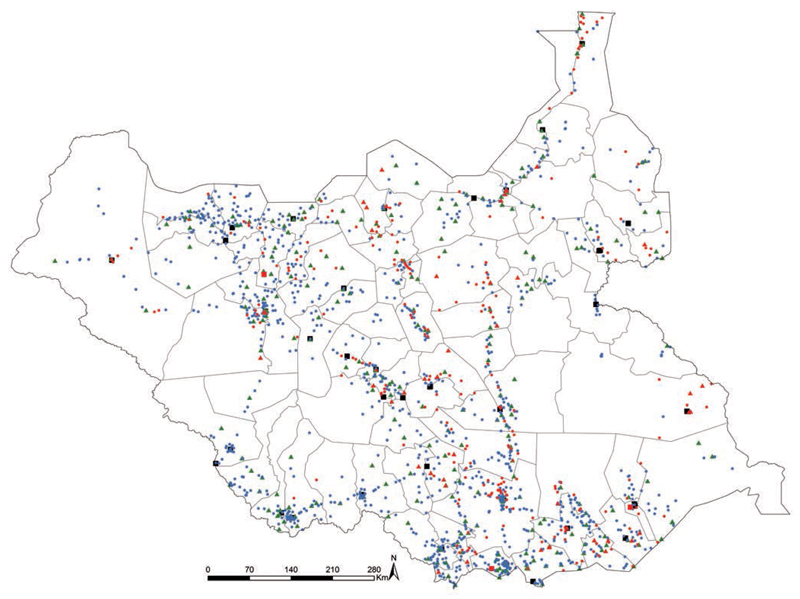
Distribution of functional (n=1453) and non-functional (n=294; shown in red) health facilities; Primary Health Care Unit (dot), Primary Health Care Centre (triangle) and Hospital (square).

After careful comparisons of health facilities’ spatial locations and names, duplicates (70), services dedicated for military personnel (27), specialist services (18), private facilities (15) and health facilities that were reported to be non-functional (294) were removed. Those defined as non-functional ranged from 5% in Western Equatoria State to over 23% in the states of Jonglei, Lakes and Upper Nile, and nearly 33% in Unity State (Figure 3). Three hospitals had been affected by the conflict and were defined as non-functional. Out of the 1446 geo-coded public services regarded as functional around 2016, 27, 7 and 3 were county, state and tertiary hospitals, respectively, 286 were PHCCs and 1123 were PHCUs. The distribution of the functioning public sector facilities alongside those that have been closed is shown in Figure 3 with data provided in the Appendix.

Nationally, across all facility types, the facility-to-population ratio was about 1 facility offering curative services for 7947 people. Hospitals served on average 312,000 people, each PHCC 40,373 people and each PHCU 10,218 people.

### Euclidean distance

**Figure 4:**
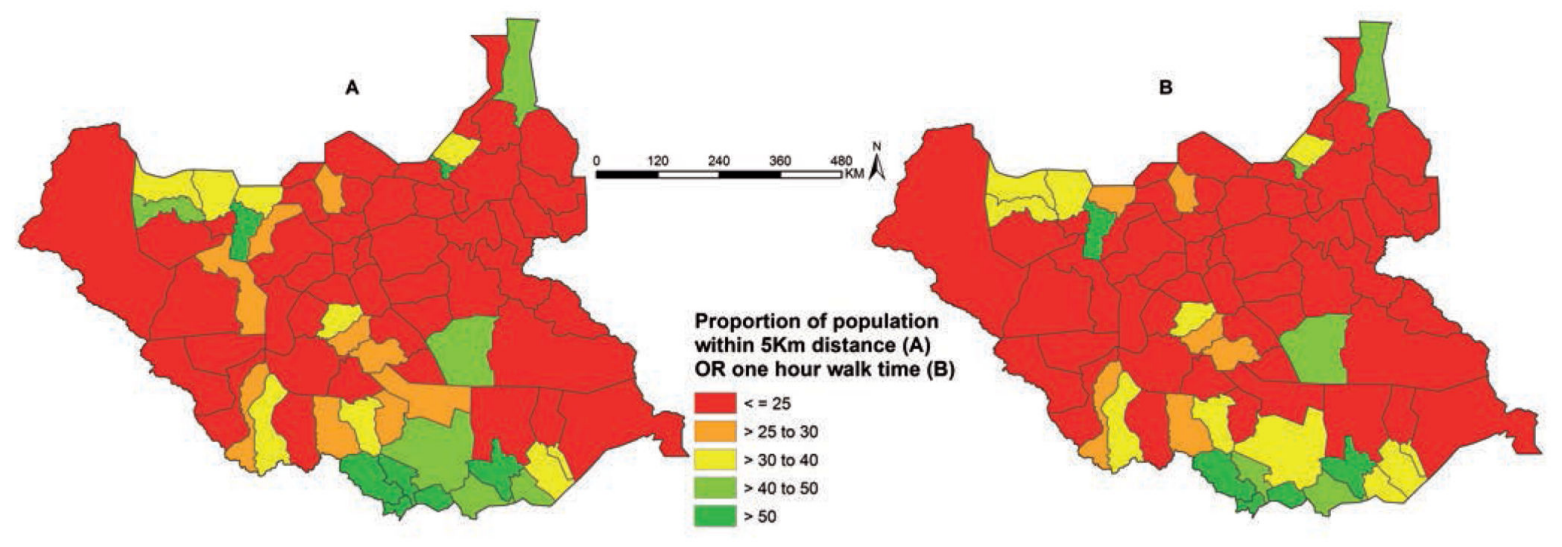
A) Proportion of population within 5 km radius of the nearest functional public health facility and (B) proportion of population within 1 hour of walking time to the nearest functional public health facility.

**Figure 5:**
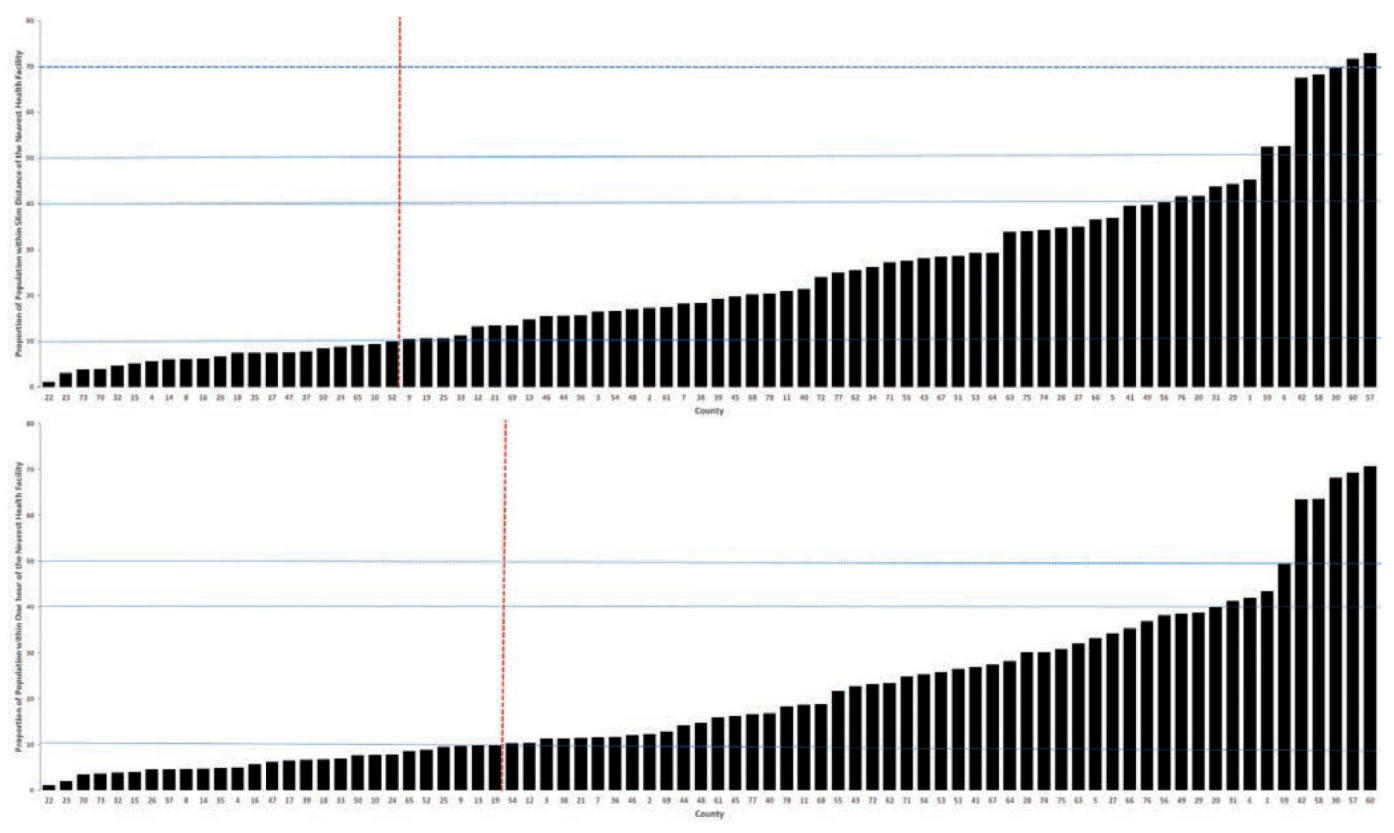
Ranked accessibility indices per county from poorest to least poor with numbers on X axis corresponding to map shown in Figure 1. Top panel is based on proportion of population within a Euclidean distance of 5 km radius of health facility; bottom panel is the proportion of the population within 1 hour walking time to nearest health facility. Dotted horizontal lines are 70% (Health Sector Development Programme target on top panel), 50%, 40% (previously described access) and 10% proportions of population within 5 km Euclidean distance (top panel) and 1-hour walking time (bottom panel). Vertical dotted line represents most *vulnerable* counties.

Only about 28.6% the population were within 5 km Euclidean distance to the nearest public health facility. Without much vehicular transport only 7.7% of the population are within 5 km of a county, state or national referral hospital. Sub-nationally, the county level aggregated data based on Euclidean distance of population to the nearest facility shows that there are only three Counties (Kajo Keji, Yei and Torit) that have reached the 70% target set by the MoH to be achieved by 2015 (Government of South Sudan Ministry of Health, 2012). 71 (91%) of the 78 counties have less than 50% of their populations within 5 km of a functioning health facility (Figures 4A and 5). 21 (27%) counties, where 10 are located in Upper Nile and Jonglei States, are most vulnerable with less than 10% of their population within a 5 km Euclidean radius of a public health facility providing clinical service (Figures 4A and 5).

### Travel time

The proportion of the population at each 1x1 km^2^ grid was assigned travel times to their nearest facility based upon a composite walking time allowing for roads access, land cover and elevation. Overall, the proportion of the population living with an estimated one hour walking time to the nearest facility was 25.7%. Over 22% of the population had a walking travel time to the nearest clinical service of more than 5 hours. Sub-nationally, only five counties had more than 50% of their populations within a 1 hour walking time to the nearest facility, 26 (33%) counties had less than 10% of their population living within 1 hour of walking to a facility (Figures 4B and 5). The 26 poorest access counties corresponded largely with those described using Euclidean distance methods (Figure 5).

## Discussion

Only 28.6% of the population are within 5 km of a public health curative service, a long way from the HSDP’s ambition of 70% (Government of South Sudan Ministry of Health, 2012). More than 74% of the population live more than 1 hour of walking away from a public health facility. The findings suggest that the situation is much worse than previously reported (WHO, 2009; Cometto *et al*., 2010; Government of South Sudan Ministry of Health, 2011, 2012; Vogt *et al*., 2011; Downie, 2012; Shabalina, 2014; World Bank, 2014). Here, high-resolution publically available data have been used to develop widely used metrics of spatial access to health services and used to describe the sub-national disparities in access. While the picture overall is one of poor access, across all metrics presented, it is worse in some specific counties (Figures 4 and 5), notably the counties of Pibor, Pochalla, Nagero, Raga and Paring. Since December 2013, when the current civil war started, there have been major disruptions of public services, affecting an already fragile health system (Berendes *et al*., 2014; Green, 2014; UN OCHA 2014b; nternational Organization for Migration, 2016) contributing to the closure of many facilities (Figure 3). Despite multilayered modelling of travel times, using more complex levels of analysis, counties with the poorest access were identified equally with simpler measures of Euclidean distance metrics (Figures 4 and 5). Approaches to modelling spatial access have grown over the last decade, inlcuding gravity models such as kernel density (Guagliardo *et al*., 2004) or two-step floating catchment area methods (Radke and Mu, 2000; Luo and Qi, 2009). However, all these depend intrinsically on the quality of input data, notably the accuracy of health service locations, population, travel routes and an understanding of travel modes. In studies whose aim is to compare relative access to care as opposed to a description of elaborate journey paths, Euclidean distance might be appropriate and can be used sub-nationally to guide effective allocation of resources to meet apparent unmet needs (Nesbitt *et al*., 2014; Gautam *et al*., 2014). As shown in the present study (Figures 4 and 5) Euclidean distance was comparable to the more complex travel time models allowing appropriate identification of vulnerable counties. There are obvious caveats to the analysis of the data assembled here. Firstly, there will be a huge uncertainty in the maps of population distribution and density used to compute the geographic coverage of health services. These have used old census input data and do not reflect the post-independence human settlement. Settlement maps have been developed by OCHA to support humanitarian assistance (UN OCHA, 2016b), but these do not have population counts and would fluctuate almost daily within the context of the current civil strife. However, more data are available than currently used by WorldPop to provide a more reliable population surface. Secondly, there is a paucity of detailed information on the travel modes populations frequently use to reach health facilities and these vary in times of conflict and precisely how much non-walking transport is available. It is also probably true that the population minority living in urban areas access services differently to those in rural areas. Finally, the assumption in the travel-time models is that people use the nearest health facility, which ignores other influences on choice of service provider (Gulliford *et al*., 2002). These combined factors, which cannot be addressed with current data, have a greater influence on the travel-time in the models developed compared to the Euclidean models.

## Conclusions

Spatial accessibility to health services in South Sudan remains very low, with an estimated 71% of the population living in areas outside 5 km, which is approximately equivalent to 1 hour from a public health facility. This low spatial accessibility is primarily a function of the inadequate distribution of health facilities, but also the inoperability of over 294 health facilities. The analysis presented here suggests that the simpler Euclidean distance models probably perform as well compared to more complex models. The metrics may be used by the South Sudan MoH to plan the distribution of resources more equitably to improve access to care and by humanitarian agencies to strategically invest in the dysfunctional counties that have suffered from conflict.

## Supplementary Material

Facilities
